# Nationwide assessment of insecticide susceptibility in *Anopheles gambiae* populations from Zimbabwe

**DOI:** 10.1186/1475-2875-13-408

**Published:** 2014-10-17

**Authors:** Nzira Lukwa, Shadreck Sande, Aramu Makuwaza, Tonderai Chiwade, Martin Netsa, Kwame Asamoa, Gonzalo Vazquez-Prokopec, Richard Reithinger, Jacob Williams

**Affiliations:** National Institute of Health Research, Harare, Zimbabwe; National Malaria Control Programme, Harare, Zimbabwe; RTI International, Harare, Zimbabwe; Malaria Branch, U.S. Centers for Disease Control and Prevention, Harare, Zimbabwe; Department of Environmental Studies, Emory University, Atlanta, GA USA; Global Health Division, International Development Group, RTI International, Suite 750, 701 13th Street, Washington D.C., 20005 USA; Faculty of Infectious Diseases, London School of Hygiene and Tropical Medicine, London, UK

## Abstract

**Background:**

The scale-up of malaria interventions in sub-Saharan Africa has been accompanied by a dramatic increase in insecticide resistance in *Anopheles* spp. In Zimbabwe resistance to pyrethroid insecticides was reported in Gokwe District in 2008. This study reports results of the first nation-wide assessment of insecticide susceptibility in wild populations of *Anopheles gambiae sensu lato* (*s.l*.) in Zimbabwe, and provides a comprehensive review of the insecticide resistance status of *An. gambiae s.l.* in southern African countries.

**Methods:**

World Health Organization (WHO) insecticide susceptibility tests were performed on 2,568 field collected mosquitoes originating from 13 sentinel sites covering all endemic regions in Zimbabwe in 2011–2012. At each site, 24-hour mortality and knock-down values for 50% and 90% of exposed mosquitoes (KD_50_ and KD_90_, respectively) were calculated for pools of 20–84 (mean, 54) mosquitoes exposed to 4% DDT, 0.1% bendiocarb, 0.05% λ-cyhalothrin or 5% malathion. Susceptibility results from Zimbabwe were compiled with results published during 2002–2012 for all southern African countries to investigate the resistance status of *An. gambiae s.l.* in the region.

**Results:**

Using WHO criteria, insecticide resistance was not detected at any site sampled and for any of the insecticide formulations tested during the malaria transmission season in 2012. Knock-down within 1 hr post-insecticide exposure ranged from 95% to 100%; mortality 24 hours post-insecticide exposure ranged from 98% to 100%. Despite the lack of insecticide resistance, high variability was found across sites in KD_50_ and KD_90_ values. A total of 24 out of 64 (37.5%) sites in southern Africa with reported data had evidence of phenotypic insecticide resistance in *An. gambiae s.l.* to at least one insecticide.

**Conclusion:**

Despite a long history of indoor residual spraying of households with insecticide, up to 2012 there was no evidence of phenotypic resistance to any of the four insecticide classes in *An. gambiae s.l.* collected across different eco-epidemiological areas in Zimbabwe. Results reinforce the need for careful monitoring over time in sentinel sites in order to detect the potential emergence and propagation of insecticide resistance as insecticidal vector control interventions in Zimbabwe continue to be implemented.

**Electronic supplementary material:**

The online version of this article (doi:10.1186/1475-2875-13-408) contains supplementary material, which is available to authorized users.

## Background

Over the past 10 years malaria interventions have been scaled-up throughout sub-Saharan Africa, including long-lasting insecticidal nets (LLINs), indoor residual spraying (IRS) of households with insecticide, as well as diagnosis and case management of malaria. This has resulted in significant reductions in disease burden and human exposure to *Anopheles* spp. mosquitoes [1]. Thus, international partnerships and economic and political support have led to the distribution of 66–145 million LLINs every year (increasing household LLIN ownership from 3% in 2000 to 56% in 2012) and coverage of approximately 8% of households in the WHO Africa Region with IRS [1]. As a consequence, malaria mortality in African countries has dropped by 49% between 2000 and 2012 [1]. The success of such vector control efforts has been, in part, due to their increased geographic coverage, and the efficacy and residual power of the various insecticide formulations employed [1, 2]. The emergence of insecticide resistance in *Anopheles* spp. is likely to be mainly a result of strong selective pressure imparted by the scale-up of interventions and the massive use of agrochemicals (e.g., [3–11]), and is now one of the major challenges affecting the future impact and sustainability of current vector control interventions in sub-Saharan Africa.

Four classes of chemicals are recommended for use in IRS or on LLINs: carbamates (e.g., bendiocarb, propoxur), organochlorines (e.g., dichlorodiphenyltrichloroethane [DDT]), organophosphates (e.g., malathion, pirimiphos methyl) and pyrethroids (e.g., deltamethrin, λ-cyhalothrin); notably, pyrethroids are the only insecticides currently used in LLINs recommended by WHO [12]. Single, multiple and cross-resistance of *Anopheles* spp. to insecticides is now considered widespread throughout sub-Saharan Africa, particularly for pyrethroid insecticides [4, 13–15]. Although increases in the allelic frequency of *Anopheles* spp. genes known to confer resistance to pyrethroids have been linked to the increased coverage of LLINs and IRS [4], the effect of such resistance on the efficacy of these interventions can be variable [16, 17]. Given the dramatic increase in insecticide resistance in malaria-endemic countries, the World Health Organization (WHO) and Roll Back Malaria partnership launched the Global Plan for Insecticide Resistance Management (GPIRM) in 2012 [16]. A key premise of the GPIRM is that insecticide resistance management must be done pre-emptively on a country-by country basis, continuously and across representative geographic areas with different histories of insecticide use in both the public health as well as agricultural sector.

Malaria is a major health problem in Zimbabwe, with 45 of the country’s 62 districts considered endemic and 33 highly malarious [1]; 95% of cases are caused by *Plasmodium falciparum,* and *Anopheles arabiensis* is the primary vector. The country has a long history of vector control against *Anopheles gambiae sensu lato* (*s.l.*) dating back to the 1950s, with IRS using DDT and pyrethroids being the main pillar of malaria interventions for over 30 years [17]; LLINs have been distributed recently, with the first mass distributions of LLINs not occurring until 2008. In the past decade, the country’s economic challenges has led –at times– to interruptions of the malaria control and surveillance activities [18, 19], resulting in fragmented estimates of malaria burden and coverage of interventions. Published information on insecticide resistance in *An. gambiae s.l.* in Zimbabwe emerges from five studies [20–24]. Despite the long history of IRS in the country, only three instances of documented insecticide resistance exist. In the late 1970s, resistance to hexa-chloro benzene in the district of Chiredzi [21] led to its replacement by DDT. In 2002, resistance to DDT [23] and in 2008 resistance to a pyrethroid (permethrin) [24] was described in Gokwe District (note, Gokwe is the district with most information about malaria epidemiology and insecticide resistance in Zimbabwe, and has been split into Gokwe North and Gokwe South). Given the limited and geographically fragmented information about insecticide resistance [25], and following the selection of Zimbabwe as a focus country under the U.S. President’s Malaria Initiative (PMI), the present study describes the first nation-wide assessment of insecticide susceptibility in wild populations of *An. gambiae s.l.* The long-term goal of this nationwide assessment was to support national vector control planning and, specifically, to guide the selection of insecticides for IRS operations. The study was a collaborative effort between the Ministry of Health National Malaria Control Programme (NMCP), the National Institute of Health Research (NIHR), and the PMI-funded Integrated Vector Management Project implemented by RTI International.

## Methods

### Study area and sites sampled

**Figure 1:**
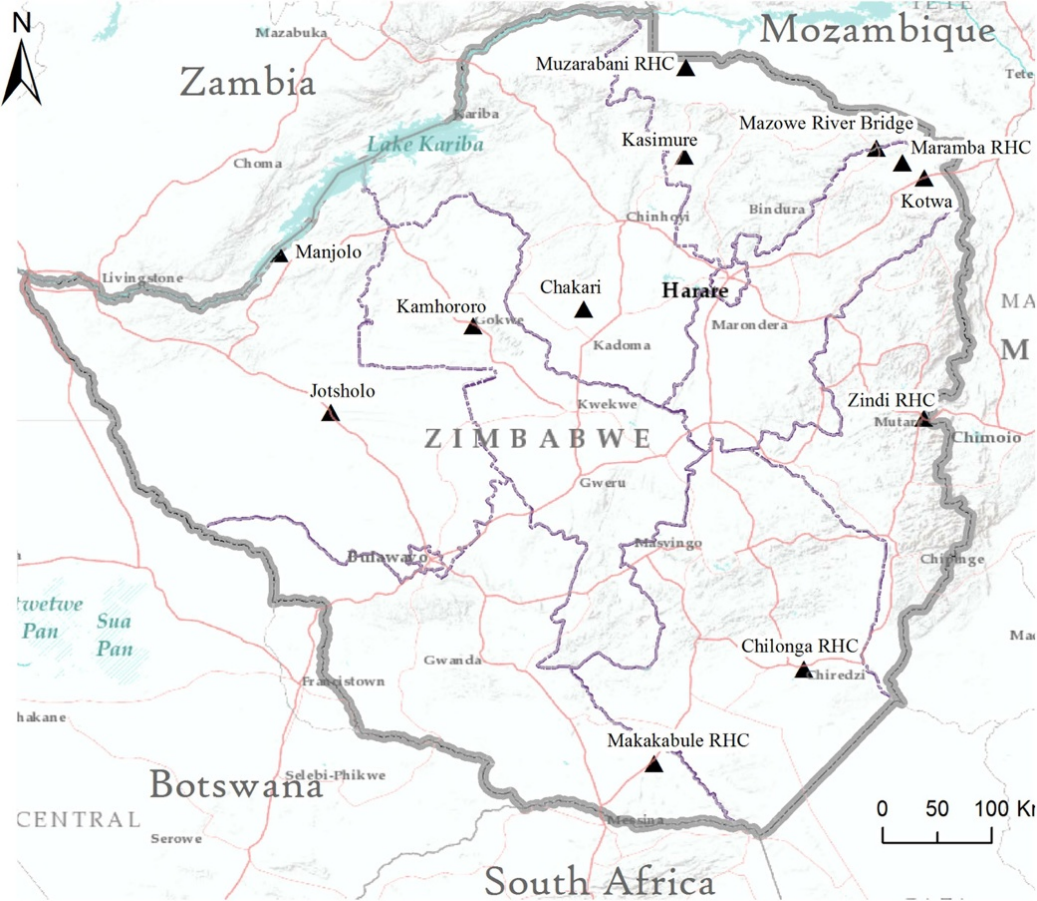
**Map of Zimbabwe indicating the geographic location of the 12 insecticide resistance monitoring sites (black triangles represent villages where susceptibility tests were performed).**

Zimbabwe is a landlocked country located in Southern Africa, with a population of ~12 million inhabitants. The country has a tropical climate, which is moderated by altitude (~80% of the country’s territory is located at elevations over 1,000 metres above sea level). The main malaria transmission season is during the rainy season, which extends from November to April, when the average temperature ranges between 18 and 30°C. The annual rainfall varies from less than 700 mm in Matabeleland North to more than 1,500 mm in Manicaland. For this nationwide assessment conducted between 2011 and 2012, 13 out of the 16 sites serving as NMCP entomological surveillance sites were sampled; sites were purposefully-selected rural villages and represented all malaria endemic regions within Zimbabwe (Figure 1). Prior to the susceptibility tests, crews of fieldworkers identified suitable *An. gambiae s.l.* breeding habitats from which mosquitoes would be collected, bred and utilized in insecticide susceptibility tests.

### Insecticide susceptibility assays

At each sentinel site, mosquito larvae were collected from breeding sites using 350 ml larval scoops (Bioquip, Gardena, CA, USA). Collected larvae were identified morphologically to species using proprietary keys [26], and reared following previously described methods [27]. *An. gambiae s.l.* larvae were separated into individual rearing bowls, and emerged adults were kept in rearing net cages and fed with 10% sucrose solution. Three to five days post-emergence, mosquitoes were separated and used in insecticide susceptibility tests using the WHO insecticide susceptibility tests [28, 29]. A mean 54 (range, 20–84) unfed female *An. gambiae s.l.* were used for each test replicate per insecticide assessed. Twenty laboratory reared *An. gambiae sensu stricto* (*s.s*.) (Kisumu strain) from an established colony in Harare were used as negative controls for each insecticide tested.

Mosquitoes were placed inside holding tubes before being exposed to WHO test papers treated with either 4% DDT, 0.1% bendiocarb, 0.05% λ-cyhalothrin or 5% malathion. The mosquitoes were exposed to each insecticide for 60 min at constant ambient conditions (25 ± 2°C and 80% relative humidity). After the exposure period, mosquitoes were transferred to the observation tube of the test kit where they were supplied with a 10% sucrose solution and held for 24 hours. The number of mosquitoes knocked down was recorded every 10 minutes for a period of 60 minutes post-insecticide exposure. If 80% knock-down was not achieved after 60 min, specimens were held for an additional 20 minutes.

### Distribution of insecticide resistance in Zimbabwe and neighbouring countries

A literature search on recent (2002 – 2012) results of published insecticide resistance tests was performed using PubMed [30], ISI web of knowledge [31], IR mapper [32] and PMI’s report archive [33]. References were considered when they included the following elements: i) Countries neighbouring Zimbabwe (i.e., Mozambique, Zambia, Botswana, South Africa, Malawi, Namibia); ii) mosquito species identified as *An. gambiae s.l.,* or *An. arabiensis*; iii) insecticide applied belonging to one of the four main classes used in malaria control (i.e., pyrethroids, organochlorines, organophosphates or carbamates). A comprehensive database of published reports of insecticide resistance in anopheline mosquitoes was recently described by Knox *et al*. [25] – it showed the scarcity of data on mechanisms of resistance in the region and outlines gaps in available data for some countries, including Zimbabwe.

### Data analysis

The WHO criteria for defining mosquito resistance to insecticides was followed [16, 34], with resistance, tolerance (or ‘suspected’ resistance), and susceptibility considered when 24-hour mortality was <90%, between 90 and 97%, and between 98 and 100%, respectively. The KD_50_ (i.e. time, in minutes, required to achieve the knock-down of 50% of the mosquitoes) and KD_90_ (i.e. time, in minutes, required to achieve the knock-down of 90% of the mosquitoes) were calculated using standard probit analysis [34]. Non-parametric Mann–Whitney U tests were performed to compare KD_50_ and KD_90_ values between field tests and laboratory controls.

A total of 159 insecticide susceptibility records for the mosquito species and insecticide types listed were obtained for a total of 64 sites located in Zimbabwe and neighbouring countries. Data was integrated into a Geographic Information System (ArcGIS 10.1; ESRI, Redlands, CA) to generate maps describing the resistance status of *An gambiae s.l*. and, specifically, *An arabiensis* (using WHO criteria) to the four insecticide classes.

## Results

### Insecticide susceptibility assays

A total of 2,568 *An. gambiae s.l.* were used in 48 susceptibility tests at 13 sentinel sites (Table 1). Using WHO criteria, no signs of insecticide-induced resistance were detected at any site and for any of the insecticides tested. Knock-down within one hour post-insecticide exposure ranged from 95% to 100%, whereas mortality at 24 hours post-insecticide exposure ranged from 98% to 100% (Table 1). Knock-down and mortality values of the *An. gambiae s.s.* reference colony were both 100% (N = 20 mosquitoes per insecticide).

**Table 1:** **Knock-down times and percent mortality 24 hours after a 1-hour exposure to the WHO diagnostic dose of insecticide in 13** ***Anopheles gambiae*** **s** ***.l.*** **populations from Zimbabwe in 2012**

Site (District)	Insecticide	N	% Knockdown at 60 min	% Mortality at 24 hrs
Mazowe River Bridge (Rushinga)	DDT (4%)	80	100	100
	Bendiocarb (0.1%)	80	100	100
	λ-cyhalothrin (0.05%)	80	100	100
	Malathion (5%)	80	100	100
Muzarabani RHC (Centenary)	DDT (4%)	80	100	100
	Bendiocarb (0.1%)	80	100	100
	λ-cyhalothrin (0.05%)	80	100	100
	Malathion (5%)	80	100	100
Zindi RHC (Mutasa)	DDT (4%)	ND	ND	ND
	Bendiocarb (0.1%)	ND	ND	ND
	λ-cyhalothrin (0.05%)	20	100	100
	Malathion (5%)	ND	ND	ND
Chilonga RHC (Chiredzi)	DDT (4%)	40	100	100
	Bendiocarb (0.1%)	40	100	100
	λ-cyhalothrin (0.05%)	40	100	100
	Malathion (5%)	40	100	100
Makakabule RHC (Beit Bridge)	DDT (4%)	40	100	100
	Bendiocarb (0.1%)	40	100	100
	λ-cyhalothrin (0.05%)	40	100	100
	Malathion (5%)	20	100	100
Jotsholo (Lupane)	DDT (4%)	20	95	100
	Bendiocarb (0.1%)	20	100	100
	λ-cyhalothrin (0.05%)	20	100	100
	Malathion (5%)	20	100	100
Manjolo (Binga)	DDT (4%)	20	100	100
	Bendiocarb (0.1%)	20	100	100
	λ-cyhalothrin (0.05%)	20	100	100
	Malathion (5%)	20	100	100
Kamhororo (Gokwe South)	DDT (4%)	80	100	100
	Bendiocarb (0.1%)	80	100	100
	λ-cyhalothrin (0.05%)	80	100	100
	Malathion (5%)	80	100	100
Kotwa (Mudzi)	DDT (4%)	80	100	100
	Bendiocarb (0.1%)	80	100	100
	λ-cyhalothrin (0.05%)	80	100	98
	Malathion (5%)	80	100	100
Maramba RHC (Uzumba Maramba Pfungwe)	DDT (4%)	80	100	100
	Bendiocarb (0.1%)	40	100	100
	λ-cyhalothrin (0.05%)	80	100	100
	Malathion (5%)	40	100	100
Burma Valley Clinic (Mutare)	DDT (4%)	40	100	100
	Bendiocarb (0.1%)	40	100	100
	λ-cyhalothrin (0.05%)	60	100	100
	Malathion (5%)	42	100	100
Kasimure (Hurungwe)	DDT (4%)	20	100	100
	Bendiocarb (0.1%)	20	100	100
	λ-cyhalothrin (0.05%)	20	100	100
	Malathion (5%)	ND	ND	ND
Chakari (Sanyati)	DDT (4%)	84	100	100
	Bendiocarb (0.1%)	80	99	99
	λ-cyhalothrin (0.05%)	80	100	99
	Malathion (5%)	82	100	100

ND, not done; RHC, rural health centre.

**Figure 2:**
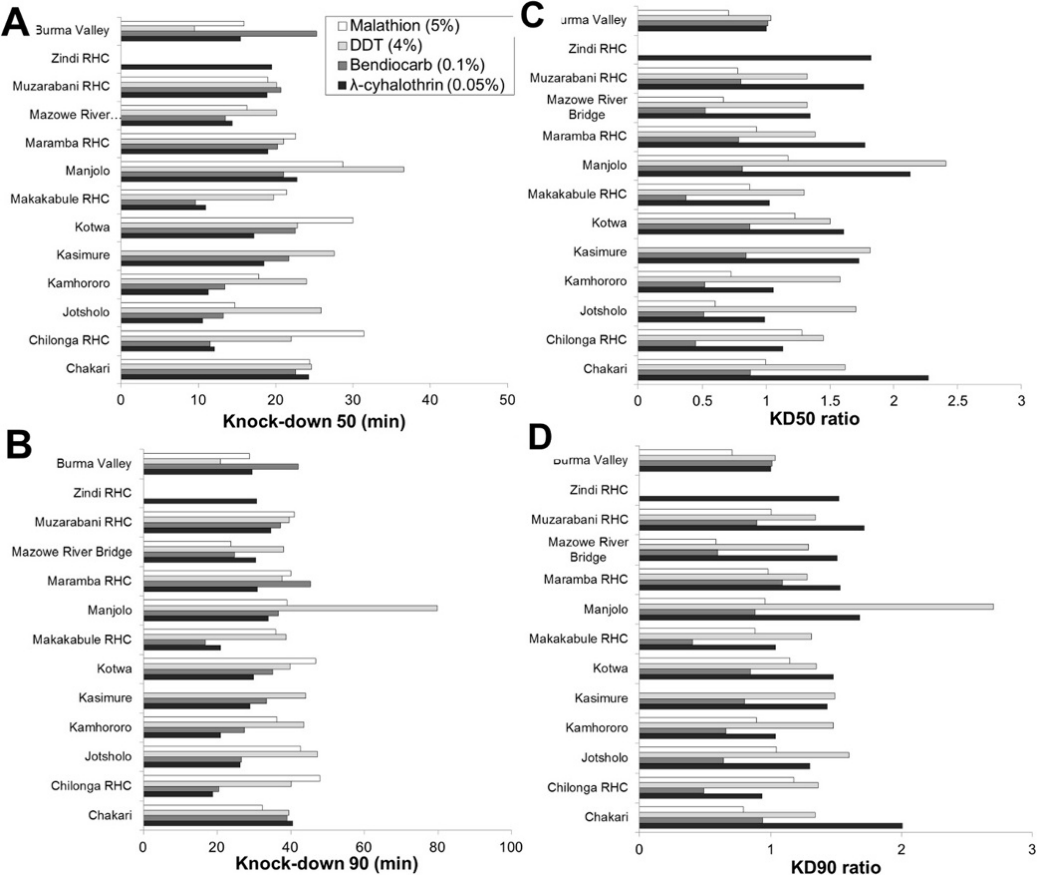
**Knock-down effect of different insecticides on** ***An.gambiae*** **s.l. mosquitoes from 13 sentinel sites in Zimbabwe. (A)** KD_50_, **(B)** KD_90_, **(C)** KD_50_ ratio, **(D)** KD_90_ ratio. KD ratios are calculated as the ratio of KD of each insecticide at each village and the KD of the reference colony.

The median KD_50_ [interquartile range] for each insecticide tested was 22.4 [20.0-26.2] min for DDT, 20.8 [13.4-22.1] min for bendiocarb, 15.9 [11.3-18.5] min for λ-cyhalothrin and 21.4 [17.0-27.7] min for malathion. Values were not significantly different from the median KD_50_ values obtained for the *An. gambiae s.s.* reference colony (15.2 min for DDT, 25.8 min for bendiocarb, 10.7 min for λ-cyhalothrin and 24.5 min for malathion) (Mann Whitney U =1.3 ; *P* > 0.05 ) (Figure 2A). A similar trend in values and lack of statistically significant difference with the reference colony was observed for KD_90_ (Figure 2B).

For each insecticide, variability in KD_50_ and KD_90_ values was observed across study sites (Figure 2A and B). KD_50_ values ranged 15.5-36.6 min for DDT, 9.6-25.3 min for bendiocarb, 9.5-26.8 min for λ-cyhalothrin and 14.7-31.4 min for malathion (Figure 2A). KD_90_ values ranged 29.6-79.8 min for DDT, 16.8-44.8 min for bendiocarb, 18.9-40.6 min for λ-cyhalothrin and 23.8-48.0 min for malathion (Figure 2B). The relative effect of each insecticide on mosquitoes from each sentinel site was calculated as a KD ratio (i.e., the ratio between the mean KD and the KD of the laboratory colony), and is shown in Figure 2C and D. Whereas none of the KD_50_ and KD_90_ ratios for bendiocarb were greater than the values observed for the reference colony, KD_50_ and KD_90_ ratios for malathion were greater than the reference colony in mosquitoes samples from 4 and 3 out of 13 sites, respectively. KD ratios for DDT and λ-cyhalothrin were highest, with values up to 2.41-2.50 (KD_50_), and 2.01-2.71 (KD_90_) times higher than the reference colony. The villages of Manjolo, and Chakari presented the highest KD_50_ and KD_90_ ratios (Figure 2).

### Regional insecticide resistance

**Figure 3:**
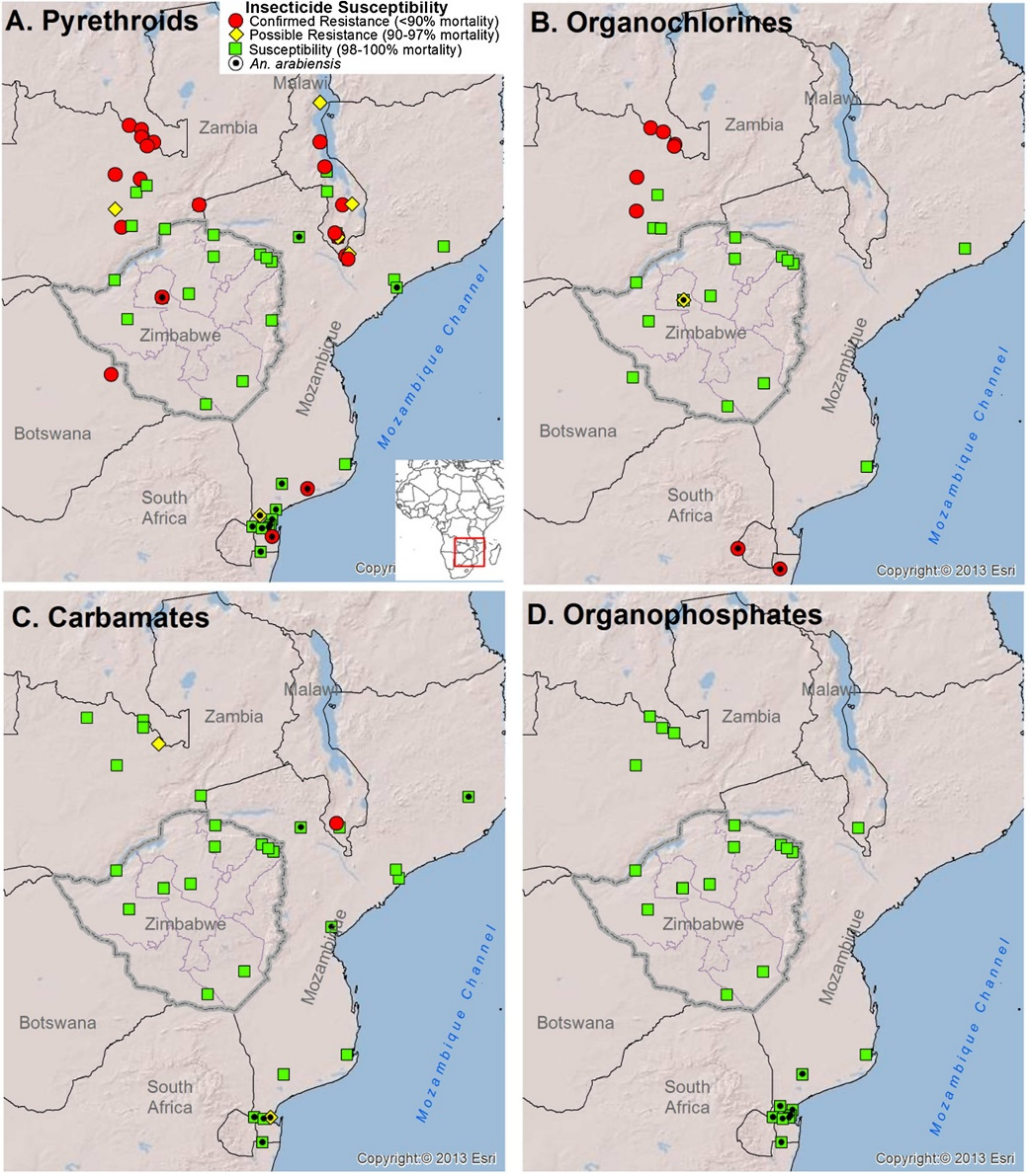
**Distribution of insecticide susceptibility in** ***Anopheles gambiae s.l.*** **and** ***Anopheles arabiensis*** **throughout Zimbabwe (this study and** [24]**) and neighbouring countries.** Different insecticide classes shown: **(A)** pyrethroids, **(B)** organochlorines, **(C)** carbamates, **(D)** organophosphates. Inset in **(A)** shows the location of the study area within Africa.

Figure 3 shows the distribution of insecticide susceptibility throughout Zimbabwe and neighbouring countries. As most data points originated from research papers, there is no uniformity in the number of insecticides and mosquitoes used at each site (see Additional file 1). Based on current WHO criteria [16, 34], a total of 24 out of 64 (37.5%) sites had evidence of insecticide resistance in *An. gambiae s.l.* for at least one insecticide formulation. Resistance to pyrethroids (mainly deltamethrin, λ-cyhalothrin and permethrin) was the most prevalent, with 20 out of 56 sites (35.7%) presenting mortality levels below 90% (Figure 3). The prevalence of resistance for the remaining insecticide classes was 8/26 (30.8%) for organochlorines, 1/34 (2.9%) for carbamates and 0/26 (0%) for organophosphates (Figure 3). When calculated by country, resistance prevalence was highest in Zambia for pyrethroids (9/14 sites, 64.3%) and organochlorines (6/9 sites, 66.7%). Malawi had the only site with confirmed resistance to carbamates (1/2 sites, 50%); no country had confirmed evidence of resistance to organophosphates (Figure 3). Botswana only had information for one site, which indicated resistance to pyrethroids, but not DDT; Namibia had no recent information on insecticide susceptibility in local *An. gambiae s.l.* populations (Figure 3).

## Discussion

Insecticide resistance among *Anopheles* spp. malaria vectors has been identified in nearly two thirds of the countries with ongoing malaria transmission [4, 17]. Given its widespread distribution and high prevalence, resistance is considered a serious challenge to the effectiveness of current vector control efforts and, therefore, sustaining gains made in reducing malaria morbidity and mortality over the past decade [1, 16]. Moreover, the extent of the problem is significantly underestimated due to the lack of sufficient country-level information: information tends to be available for a limited number of purposefully-selected sites and tends not to include all insecticide classes currently employed to control malaria vectors. With insecticidal vector control interventions being implemented at large scale in most endemic countries, the need for and use of comprehensive and routine insecticide resistance monitoring data is essential to inform the interventions’ use as well as their sustainability and effectiveness, particularly within the context of a national insecticide resistance management plan [16].

This study provides the first nation-wide assessment of insecticide susceptibility in *An. gambiae s.l.* for Zimbabwe. Relying on data from 13 sentinel sites distributed across all malaria-endemic areas in Zimbabwe, this study shows that, despite a long history of IRS applications, there is –based on WHO criteria for resistance– no evidence of insecticide resistance in *An. gambiae s.l.* to any of the four insecticides classes used for malaria control beyond published reports for Gokwe District in 2008 [24]. Although resistance levels were within susceptibility thresholds, there was significant variability across sites in KD_50_ and KD_90_ values between field mosquitoes and the laboratory reference colony. Furthermore, recent reports from Gokwe in Zimbabwe show that insecticide resistance in *An. gambiae s.l.* populations can emerge as the result of intensified insecticide pressure [23, 24]. Such results reinforce the need for careful monitoring over time in sentinel sites in order to detect the potential emergence and propagation of insecticide resistance as vector control interventions in Zimbabwe continue to be implemented (e.g., with PMI support [33]). A caveat of this study was that in some sites --due to the inability to sample enough larvae in available breeding sites for collection during the dry season-- contrary to the 2013 WHO guidelines [34] less than four replicates were carried out; however, phenotypic resistance was detected in none of the replicates. Even though no resistance to pyrethroids was detected, Zimbabwe should --in line with the GPIRM guidance [16]-- consider switching to using a non-pyrethroid insecticide for IRS in those areas targeted for LLIN distribution.

There are multiple mechanisms which can lead to insecticide resistance. Target site resistance reduces the binding of the insecticide to its site of action, increasing the survival of exposed mosquitoes [16, 35]. This is the best understood mechanism of resistance, and simple molecular diagnostic tests can detect presence of resistance or knock-down mutations [16, 35]. Metabolic resistance occurs when increased or modified activity of an enzyme system prevents the insecticide from reaching its site of action [16, 35]. The occurrence of behavioural resistance of mosquitoes due to avoidance of treated surfaces or shifts in their biting behaviour has been implicated as a potential factor driving failure of interventions; however, more research is needed before its relative role in comparison to the other physiological resistance mechanisms is known [36]. Unfortunately, this study only tested for phenotypic resistance (via susceptibility tests), and it is unknown if any resistance genes which may not yet be imparting detectable phenotypic resistance were present. In the operational context of insecticide resistance monitoring, susceptibility tests are a cost-effective tool for the assessment of susceptibility in vector populations and molecular techniques could complement bioassays in areas in which resistance is suspected or confirmed [16].

The lack of proven insecticide resistance in *An. gambiae s.l.* in this study contrasts with the data reported in neighbouring countries. The greatest number of published reports of insecticide resistance, for both DDT and pyrethroids, originate from Zambia; no country reported evidence of resistance to organophosphates. More data are needed from northern South Africa, Botswana, Namibia and western Mozambique, particularly for non-pyrethroid insecticides. Importantly, unlike the data presented in this study, not all locations had information for all insecticide classes. The development of a continental network for insecticide resistance monitoring, the African Network of Vector Resistance, represents a key component of future plans for resistance management. Selection of sites should be carefully planned to representatively cover different geographic and epidemiologic contexts. Longitudinal data from routine/ongoing surveillance activities will enable a more comprehensive understanding of how resistance develops or changes, and will allow for better establishment of correlations with vector control (and perhaps with agricultural) activities. Furthermore, the implementation of novel and open access mapping platforms such as IR mapper [25, 32] will prove essential for rapid dissemination of results and evaluation of current and future insecticide application strategies under national and regional insecticide resistance contexts.

Most of the knowledge about insecticide resistance in sub-Saharan countries has emerged from susceptibility tests performed as part of specific research studies. Although highly informative, such an approach has important limitations: a) in most cases, susceptibility tests are not performed against all insecticide classes, limiting the assessment of the status of cross and multiple resistance; b) geographic coverage is highly fragmented, affecting the ability to infer the national or sub-national status of insecticide resistance; c) tests are not performed on a regular basis (in most cases, tests were performed once), even though resistance is known to vary seasonally; d) there is limited standardization in how mosquitoes are obtained to perform tests (while the guidelines promote the use of adults emerged from field-collected larvae, many studies have used adults collected from houses, with the consequence of testing few individuals and mosquitoes of different age and physiological status). The most recent guidelines for insecticide resistance management [16] promote the establishment of insecticide resistance testing that would feed into a country insecticide resistance management plan, which would itself be part of the country’s larger malaria programme operational framework. Increasing the level of knowledge of the resistance status of malaria vectors locally and exploiting such information fully is a must for improved programmatic decision-making, and to ensure continued impact of implemented vector control interventions on malaria morbidity and mortality.

## Electronic supplementary material

Additional file 1: **Description of data sources used to generate the regional susceptibility maps presented in Figure** 3 **of the manuscript.** (XLSX 23 KB)
